# A critique of the Dispositional Flow Scale-2 (DFS-2) and Flow State Scale-2 (FSS-2)

**DOI:** 10.3389/fpsyg.2022.992813

**Published:** 2022-10-05

**Authors:** Joy Lee-Shi, Robert G. Ley

**Affiliations:** Department of Psychology, Simon Fraser University, Burnaby, BC, Canada

**Keywords:** critical analysis, state-trait, flow conceptualization, Dispositional Flow, state flow

## Introduction

Csikszentmihalyi (1975) was the first to describe the nine specific dimensions of flow, in his landmark book *Beyond Boredom and Anxiety* (Csikszentmihalyi, 2012; Rheinberg, 2012). Csikszentmihalyi's first studies in this area were based on interviews of rock-climbers and artists, who are known to persistently work with indifference to hunger, pain, and fatigue (Csikszentmihalyi, 1975; Getzels and Csikszentmihalyi, 1976). The initial academic and public reception for *Beyond Boredom and Anxiety* (1975) was lukewarm, albeit, this tepid response was during the dominance of the empirically experimental school of thought, wherein the subjective, phenomenological approach was disparaged (Csikszentmihalyi, 1975). Despite this negativity toward the subjective, interest in flow within academia grew, and eventually, was picked up by sports psychologists studying optimal experience and sports performance enhancement (Csikszentmihalyi, 2012; Nakamura and Csikszentmihalyi, 2020). These studies on optimal experience have contributed toward the current operationalization of flow, where nine dimensions of flow fall into two categories: conditions and subjective experiences.

In order for a flow experience to occur, the following conditions should be present to some degree: (i) *Challenge-Skill Balance*: “I was challenged but I believed my skills would allow me to meet the challenge”; (ii) *Clear goals*: “I knew clearly what I wanted to do”; and (iii) *Unambiguous feedback*: “I was aware of how well I was doing.” Then, during flow, individuals will often experience the following states: (i) *Autotelic experience*: “I loved the feeling of what I was doing, and want to capture this feeling again”; (ii) *Total concentration*: “It was no effort to keep my mind on what was happening”; (iii) *Sense of control*: “I felt like I could control what I was doing”; (iv) *Loss of self-consciousness*: “I was not concerned with how others may have been evaluating me”; (v) *Transformation of time*: “The way time passed seemed to be different from normal”; and finally, (vi) *Action-awareness merging*: “I did things spontaneously and automatically without having to think”. These examples are selected items from the Long Flow State Scale 2 (Jackson et al., 2010).

Recently, many approaches to measuring and studying flow dimensions have emerged. Recent studies have even taken a more objective approach and found empirical evidence of the flow experience. Some have identified behavioral or gestural markers, through a combination of quantitative and qualitative methods (de Manzano et al., 2010). A rare few have even found identifiers of flow through psychophysiological approaches (van der Linden et al., 2021). However, the most popular method continues to be the use of standardized scale questionnaires, such as Jackson et al.'s (2010) Flow Scales from the *Flow Manual* [i.e., the Flow State Scale 2 (FSS-2) and the Dispositional Flow Scale 2 (DFS-2)]. These two scales are a common choice of measurement, due to Csikszentmihalyi's foundational definition of flow, the large body of literature supporting its reliability and validity (see: Kawabata et al., 2008; Jackson et al., 2010; Riva et al., 2017; etc.,), and the distinction of state and trait.

According to the *Flow Manual* (Jackson et al., 2010), the DFS-2 assesses the “individual difference aspect to flow… [that] will remain fairly stable over a long time frame” and the FSS-2 is a “post-event assessment of flow”. There are two differences between these scales: (i) verb tense for the self-statements—past- *vs*. present- tense; and (ii) participants are assessed in terms of frequency (e.g., never, always) or veracity (e.g., disagree, strongly agree). There are 36 items in each scale, and the items are nearly identical between the two scales aside from the framed perspective of the instructions and items. The nine dimensions of flow are measured in both, and each dimension is measured by four separate items.

## Discussion

It is likely that the FSS-2 and DFS-2 may not strictly assess flow as one may expect them to. While they have been proven to be reliable and consistent with Csikszentmihalyi's (1975) definition of flow (Jackson et al., 2010), questions regarding the validity of specific dimensions have been raised. In addition to this, there are major limitations concerning flow score interpretation, the scoring procedure of scales, as well as the conceptualization and assessment of their construct across literature.

There are two approaches to scoring and interpreting flow scores (Jackson et al., 2010). First is calculating the sum of the nine averaged dimension scores, in order to obtain an overall flow score. This strategy implies that there is a range of intensity in the flow experience. One can have “low flow” or “high flow” in the same manner that one can have low or high anxiety. In fact, according to the flow literature, the intensity of flow experiences can be placed on a continuum, in relation to the duration and complexity of the task at hand (Lavoie et al., 2022). According to the *Flow Manual* (Jackson et al., 2010), low item scores indicate a “substantively less flow-like nature” in the assessed dimension, while high item scores indicate the opposite. However, these flow scores may be interpreted as: (a) there is a range in the intensity of flow experiences; or (b) there is a range in the probability of flow experiences. Depending on the researcher, a high flow score may be interpreted as an intense flow experience or as an experience that is very likely to be flow. Another problem arises in that two identically high scores may consist of different combinations of extremely high dimensional averages and close to zero averages.

The second recommended scoring is calculating the average score for each of the nine dimensions to create a multidimensional profile; this approach is recommended due to the construct of the flow concept. This also resolves the issue of obtaining two identical scores from significantly different dimensional values. While this strategy provides in-depth data about the experience of flow and its components, the “sum of its parts” may not equate the whole, if the goal was to understand the experience itself (Sabar, 2013). For instance, reviewing a list of baking ingredients and measurements may not give an accurate idea of the intended product—it could be interpreted as a cake, a muffin, pancakes, etc. In following with this metaphor, it is possible that certain individuals may not experience the same level or intensity of flow with the same amount of ingredients. Additionally, this assumes that all nine dimensions are valid within the contexts of the tasks at hand.

There are several concerns about the validity of certain flow dimensions, within and between the state and trait scales. Given that items are nearly identical except for verb tense and instruction time-frame, the scales are in fact self-reports that: (i) assess the memory of a recent flow experience and (ii) assess the memory of overall flow experiences. According to Nakamura and Csikszentmihalyi (2020), the flow experience is a product of an individual's innate characteristics and the external environmental factors. Consequently, it is unlikely that the nine dimensions of flow are equally influenced by internal and external variables, as each dimension may vary in terms of how much it proportionally contributes to flow as a state or as a trait. For instance, the autotelic dimension has often been interpreted as a dispositional trait characterization. Compared to this however, the unambiguous feedback dimension is more closely associated with a state. Clearly, there is much to be examined here, as there are large bodies of literature addressing this concept in other mental phenomena, such as state and trait anxiety, state and trait efficacy, and so forth.

In terms of state-trait conceptualization, flow and self-efficacy are very similar. Self-efficacy can be regarded as a state or a trait; however, this is just one common approach. Self-efficacy can also be organized into three situational contexts: *general* or *trait-like*, which refers to a general stable belief in one's capability in completing a task; *domain-specific*, which often relates to the ability to manage health/illness situations; and finally, *task-specific*, which refers to context specific behavior (van Diemen et al., 2020). Note that d*omain-specific* and *task-specific* are two categories that originate from state self-efficacy. Unfortunately, distinctions between these three aspects still require further research and clarification (van Diemen et al., 2020).

Perhaps, current understandings of flow can be advanced through inspiration from already existent construct operationalizations such as has occurred with state self-efficacy. Or perhaps, further efforts of distinguishing between state and trait flow may prove to be fruitless, as most psychological constructs are neither one or the other, but rather of a combination of both state and trait attributes (Geiser and Simmons, 2021). Thus, a more productive direction of research may be to consider and study flow within a completely different framework—such as that of a construct lying on a number of continua, i.e., intensity, duration, complexity (Lavoie et al., 2022).

Researchers have also challenged the inclusion of specific dimensions within the flow conceptualization. For instance, Lovoll and Vitterso (2014) argued that the requirement of *Challenge-Skill Balance* dimension, long regarded as an essential condition of flow, should instead be at an imbalance. They found that a perfect balance can lead to boredom or disinterest, and that a high challenge higher or lower than high skills is more strongly associated with flow. However, that study was based on students on a recent ski trip. Studies have found that dimensional validity issues are found especially in music students and athletes (Jackson et al., 2010; Sinnamon et al., 2012). These dimensions include the following: *time transformation, loss of self-consciousness*, and *clear goals*. Interestingly, validity issues for *clear goals* only apply for “elite athletes.” No statements can be made about music students and musicians in general, however, as the majority of flow and music research literature contains a limited range of participant demographics, i.e., schoolchildren and youths.

These issues give light to a major concern regarding the conceptualization and operationalization of flow across the research literature: in short, there is great need for theoretical integration. For instance, a conceptual analysis found 24 distinct operationalized constructs of flow in a total of 42 articles (Abuhamdeh, 2020). It is very clear that there is a large number of flow scales and definitions (i.e., Ghani and Deshpande, 1994; Rheinberg et al., 2003; Pearce et al., 2005; Kiili, 2006; Schüler, 2007; Hung et al., 2015). Some constructs that differ from the DFS-2 and FSS-2 dimensions include: absorption by activity, feelings of frustration, and enjoyability. Despite the similarities to the DFS/FSS-2 dimensions of concentration and autotelic experience, they are not identical in scope. It should be noted that the DFS-2 and FSS-2 were selected for the present discussion due to their roots in the original flow conceptualization—Csikszentmihalyi's conceptualization can be considered as the “default” (Abuhamdeh, 2020). Within the conceptual analysis, the author concluded that there are three types of conceptual or operational inconsistencies throughout the flow literature: (i) Is flow continuous or discrete?; (ii) Is enjoyability inherent to flow or not?; and (iii) Is flow dependent or distinct from its antecedents/conditions?

The DFS-2 and FSS-2 may be considered to be continuous; however, a continuum of “probably not flow” to “probably flow” or one of “0% properties of flow” to “100% properties of flow” does not give as much significance as a continuum that describes variation in flow intensity. Besides this questionable quantitative representation of the flow construct, there are conceptual issues. Not all flow scales contain the same sort of dimensions; in addition to this, many flow scales contain varying quantities of dimensions.

In many cases, while dimensional constructs are similar and near-identical between certain operationalizations, different terminologies may be used. For instance, the following terms are used in some flow studies: *fluency of performance, feelings of frustration*, and *absorption by activity* (see: Rheinberg et al., 2003; Rachmatullah et al., 2021). Despite the difference in terminology, the first two may be associated with *action-awareness merging* and *autotelic experience*, while the last may be used as another word for flow. Thus, the following criticism about the lack of “theoretical integration,” borrowed from an article on motivational theories, is quite accurate.

[The] theories in psychology are dispersed across a whole field of historical orientations, overlapping concepts, and differentially-related constructs. This diffusion is manifested as a “proliferation” of terms and constructs which range in nomenclature… [with] idiosyncratic vocabulary using different words for the same concept and the same word for different concepts… (Duncan et al., 2021).

Though it is possible that the componential approach of measuring flow as a multi-dimensional state-trait variable, i.e., the DFS-2 and FSS-2, may have been a step toward theoretical integration, it is often seen as just another set of scales to choose from. Sometimes, it is treated as a collection of constructs for custom handpicking (see: Lee, 2005; Bassi and Delle Fave, 2011). Many studies are often just interested in examining various distinct theories on an individual basis, rather than in exploring the possible relationships or connections between them.

In conclusion, it is imperative that flow research and its accumulating literature can concretely answer the questions: “What is flow?” and “Does this [method] measure flow?”; unfortunately with the current limitations in research and literature, one can only state “it depends… it's complicated” and “probably.” Thus, critical analyses, meta-analyses, network analyses and qualitative studies may be invaluable tools in arriving at concrete answers to these sorts of questions. In addition to this, neurocognitive research approaches may provide further information that corroborates current foundational elements of flow, as seen in mindfulness/meditation research (see: Raffone and Srinivasan, 2017).

## Author contributions

JL-S: main writer. RL: advisor and editor. Both authors contributed to the article and approved the submitted version.

## Conflict of interest

The authors declare that the research was conducted in the absence of any commercial or financial relationships that could be construed as a potential conflict of interest.

## Publisher's note

All claims expressed in this article are solely those of the authors and do not necessarily represent those of their affiliated organizations, or those of the publisher, the editors and the reviewers. Any product that may be evaluated in this article, or claim that may be made by its manufacturer, is not guaranteed or endorsed by the publisher.

## References

[B1] Abuhamdeh S. (2020). Investigating the “Flow” experience: key conceptual and operational issues. Front. Psychol. 11:158. 10.3389/fpsyg.2020.00158 32116954 PMC7033418

[B2] Bassi M. Delle Fave A. (2011). Optimal experience and self-determination at school: joining perspectives. Motiv. Emot. 36, 425–438. 10.1007/s11031-011-9268-z

[B3] Csikszentmihalyi M. (1975). Beyond Boredom and Anxiety, 1st Edn. San Francisco, CA: Jossey-Bass Publishers.

[B4] Csikszentmihalyi M. (2012). “Foreward,” in Advances in Flow Research, ed S. Engeser (New York, NY: Springer), v–vii.

[B5] de Manzano Ö. Theorell T. Harmat L. Ullén F. (2010). The psychophysiology of flow during piano playing. Emotion 10, 301–311. 10.1037/a0018432 20515220

[B6] Duncan C. E. Kim M. Baek S. Wu K. Y. Y. Sankey D. (2021). The limits of motivation theory in education and the dynamics of value-embedded learning (VEL). Educ. Philos. Theory, 54, 618–629. 10.1080/00131857.2021.1897575

[B7] Geiser C. Simmons T. (2021). Are Psychological Constructs Traits or States? A Preliminary Review of LST Studies. Presentation for the conference of European Association of Psychological Assessment Digital Event.

[B8] Getzels J. W. Csikszentmihalyi M. (1976). The Creative Vision: A Longitudinal Study of Problem Finding in Art. New York, NY: John Wiley & Sons.

[B9] Ghani J. A. Deshpande S. P. (1994). Task characteristics and the experience of optimal flow in human—computer interaction. J. Psychol. 128, 381–391. 10.1080/00223980.1994.9712742

[B10] Hung C.-Y. Sun J. C.-Y. Yu P.-T. (2015). The benefits of a challenge: student motivation and flow experience in tablet-PC-game-based learning. Interactive Learn. Environ. 23, 172–190. 10.1080/10494820.2014.997248

[B11] Jackson S. Eklund R. Martin A. (2010). The FLOW Manual. Mind Garden Inc. Available online at: https://www.mindgarden.com/flow-scales/467-flow-manual.html. 10.1037/t06469-000 (accessed October 24, 2021).

[B12] Kawabata M. Mallett C. Jackson S. (2008). The Flow State Scale-2 and Dispositional Flow Scale-2: examination of factorial validity and reliability for Japanese adults. Psychol. Sport Exerc. 9, 465–485. 10.1016/j.psychsport.2007.05.005

[B13] Kiili K. (2006). Evaluations of an experiential gaming model. Hum. Technol. Interdiscip. J. Hum. ICT Environ. 2, 187–201. 10.17011/ht/urn.2006518

[B14] Lavoie R. Main K. Stuart-Edwards A. (2022). Flow theory: advancing the two-dimensional conceptualization. Motiv. Emot. 46, 38–58. 10.1007/s11031-021-09911-4

[B15] Lee E. (2005). The relationship of motivation and flow experience to academic procrastination in university students. J. Genet. Psychol. 166, 5–15. 10.3200/GNTP.166.1.5-15 15782675

[B16] Lovoll H. S. Vitterso J. (2014). Can balance be boring? A critique of the “Challenges Should Match Skills” hypotheses in flow theory. Soc. Indic. Res. 115, 117–136. 10.1007/s11205-012-0211-9

[B17] Nakamura J. Csikszentmihalyi M. (2020). “The experience of flow: theory and research,” in The Oxford Handbook of Positive Psychology, 3rd Edn, eds C. R. Snyder, S. J. Lopez, L. M. Edwards, and S. C. Marques (New York, NY: Oxford University Press), 279–296. 10.1093/oxfordhb/9780199396511.013.1634607296

[B18] Pearce J. M. Ainley M. Howard S. (2005). The ebb and flow of online learning. Comput. Human Behav. 21, 745–771. 10.1016/S0747-5632(04)00036-630725376

[B19] Rachmatullah A. Reichsman F. Lord T. Dorsey C. Mott B. Lester J.. (2021). Modeling secondary students' genetics learning in a game-based environment: integrating the expectancy-value theory of achievement motivation and flow theory. J. Sci. Educ. Technol. 30, 511–528. 10.1007/s10956-020-09896-8

[B20] Raffone A. Srinivasan N. (2017). Mindfulness and cognitive functions: toward a unifying neurocognitive framework. Mindfulness 8, 1–9. 10.1007/s12671-016-0654-1

[B21] Rheinberg F. (2012). “Foreward,” in Advances in Flow Research, ed S. Engeser (New York, NY: Springer), ix–xi.

[B22] Rheinberg F. Vollmeyer R. Engeser S. (2003). “Die Erfassung des Flow-Erlebens [The Assessment of Flow Experience],” in Diagnostik von Selbstkonzept, Lernmotivation und Selbstregulation [Diagnosis of Motivation and Self-Concept], eds J. Stiensmeier-Pelster and F. Rheinberg (Göttingen: Hogrefe).

[B23] Riva E. F. M. Riva G. Talò C. Boffi M. Rainisio N. Pola L.. (2017). Measuring Dispositional Flow: validity and reliability of the Dispositional Flow State Scale 2, Italian version. PLoS ONE 12:e0182201. 10.1371/journal.pone.0182201 28877167 PMC5587230

[B24] Sabar S. (2013). What's a Gestalt? Gestalt Rev. 17, 6–34. 10.5325/gestaltreview.17.1.0006

[B25] Schüler J. (2007). Arousal of flow experience in a learning setting and its effects on exam performance and affect. Z. Pädagogische Psychol. / German J. Educ. Psychol. 21, 217–227. 10.1024/1010-0652.21.3.217

[B26] Sinnamon S. Moran A. O'Connell M. (2012). Flow among musicians: measuring peak experiences of student performers. J. Res. Music Educ. 60, 6–25. 10.1177/0022429411434931

[B27] van der Linden D. Tops M. Bakker A. B. (2021). Go with the flow: a neuroscientific view on being fully engaged. Eur. J. Neurosci. 53, 947–963. 10.1111/ejn.15014 33084102 PMC7983950

[B28] van Diemen T. Craig A. van Nes I. J. W. van Laake C. Bloemen J. Stolwijk-Swuste J.. (2020). Enhancing our conceptual understanding of state and trait self-efficacy by correlational analysis of four self-efficacy scales in people with spinal cord injury. BMC Psychol. 8, 108. 10.1186/s40359-020-00474-6 33076995 PMC7574195

